# Surgeons’ and methodologists’ perceptions of utilising an expertise-based randomised controlled trial design: a qualitative study

**DOI:** 10.1186/s13063-018-2832-z

**Published:** 2018-09-06

**Authors:** Jonathan A. Cook, Marion K. Campbell, Katie Gillies, Zoë Skea

**Affiliations:** 1 Centre for Statistics in Medicine, Nuffield Department of Orthopaedics, Rheumatology and Musculoskeletal Sciences, University of Oxford, Windmill Rd, Oxford, OX3 7LD UK; 2 Surgical Intervention Trials Unit, Nuffield Department of Orthopaedics, Rheumatology and Musculoskeletal Sciences, University of Oxford, Botnar Research Centre, Windmill Rd, Oxford, OX3 7LD UK; 3 Health Services Research Unit, University of Aberdeen, Health Sciences Building, Foresterhill, Aberdeen, AB25 2ZD UK

## Abstract

**Background:**

Randomised controlled trials (RCTs) are widely recognised to be the most rigorous way to test new and emerging clinical interventions. When the interventions under study are two different surgical procedures, however, surgeons are required to be trained and sufficiently proficient in the different surgical approaches to take part in such a trial. It is often the case that even where surgeons can perform both trial surgical procedures, they have a preference and/or have more expertise in one of the procedures. The expertise-based trial design, where participating surgeons only provide the procedure in which they have appropriate expertise, has been proposed to overcome this problem. When expertise-based designs should be best used remains unclear; such approaches may be more suited to addressing specific questions. The aim of this qualitative study was to improve understanding about the range of views that surgeons and methodologists have regarding the use of the expertise-based RCT design.

**Methods:**

Twelve individual interviews with surgeons and methodologists with experience of surgical trials were conducted. Interviews were semi-structured and conducted face-to-face or by telephone. Interviews were audio-recorded, transcribed and analysed systematically using an interpretive approach.

**Results:**

Both surgeons and methodologists saw potential advantages in the expertise-based design particularly in terms of surgeons’ participation and in trials where the procedures being evaluated were significantly different. The main disadvantages identified were methodological (e.g. the potential for surgeons carrying out one of the trial procedure being systematically different) and operational (e.g. the need to ‘transfer’ patients between surgeons with potential consequences for the surgeon/patient relationship).

**Conclusion:**

This study suggests that the expertise-based trial design has significant potential to increase surgeon participation in trials in some settings. In other settings the standard design was generally seen as the preferable design. Particularly suitable conditions for an expertise-based design include those where the surgical procedures under evaluation are substantially different, where they are routinely delivered by different health professionals/surgeons with clear proficiencies in each; and contexts in which a multiple-surgeon model is in use and trust between the patient and surgeons can be suitably protected. The standard design was seen by most participants as the default design. Several logistical and methodological concerns remain to be addressed before the expertise-based design is likely to be more widely adopted.

**Electronic supplementary material:**

The online version of this article (10.1186/s13063-018-2832-z) contains supplementary material, which is available to authorized users.

## Background

Randomised controlled trials (RCTs) are widely recognised to be the most rigorous way to test new and emerging clinical interventions. While many surgeons accept the need in principle for RCTs, some surgeons may be reluctant to participate in a standard RCT if they have to perform a surgical procedure in which they have less experience/believe is less effective than the alternative treatment in their hands. As surgery is a craft specialty, where training and skill is honed through practice, it is often the case that even where surgeons can perform both trial surgical procedures, they have a preference for, and/or have more expertise in, one procedure than the other. When the surgical procedures under evaluation differ substantially, surgeons may only routinely conduct one of the trial procedures. This creates a number of potential issues for surgeons participating in a RCT evaluating two surgical procedures; some surgeons may be less willing to participate if they will have to deliver an operation they are less comfortable with, they may be less willing to facilitate patient recruitment as they may be uncertain about the manner in which the procedure will be delivered, and where they do deliver the procedure they are less confidence in, they may be less likely to fully comply (i.e. there may be more conversions to the preferred procedure). Furthermore, the newer and less familiar procedure might be argued to not be assessed in its optimal form as they are learning the procedure during the running of the trial.

**Fig. 1:**
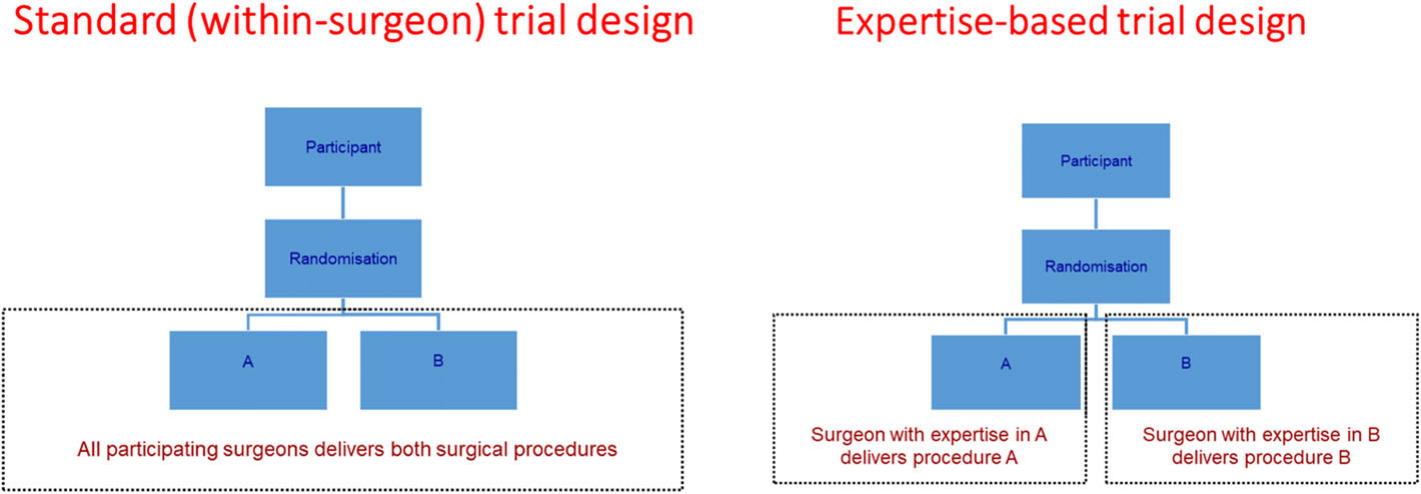
Standard and expertise-based two-arm trial designs

An *expertise-based* approach to trial design, where participating surgeons only provide the procedure in which they have greatest expertise has been proposed to overcome these problems [1]. This differs from a standard two-arm RCT in which patients are randomly allocated to receive either intervention A or intervention B (see Fig. 1). When the intervention under study is a new drug (and is being compared against a comparator drug or placebo) physicians generally require the same skill set to provide both interventions. However, when the interventions under study are two different surgical procedures, surgeons are required to be trained and to be sufficiently proficient in the different surgical approaches to take part in such a trial – delivery of the interventions can be said to be *within-surgeon*. This is classed as the standard surgical RCT design. Under an expertise-based trial design, a patient is randomly allocated to receive a specific trial surgical procedure; the allocated procedure is then delivered by an expert in the specific surgical procedure. Participating surgeons are not required to deliver both surgical procedures.

Purported benefits of the expertise-based design include increased surgeon participation and compliance with allocated intervention, addressing the learning curve effect along with desirability from a patient perspective in terms of the procedure being conducted by an ‘expert’ and according to the surgeon’s usual care and preference [1]. While such a design is not new [2], the profile of the design is increasing and its use appears to be becoming more common [3, 4]. For some comparisons, such as surgery versus non-operative management (e.g. surgical repair versus physiotherapy for the management of a rotator cuff tear), where each intervention is carried out by a different health professional) an expertise-based trial design is often the default design, although not typically termed as such. Expertise-based designs have, however, been criticised on a number of grounds and many methodological considerations have been highlighted (e.g. the impact upon sample size) [4]. In particular how ‘expertise’ should be defined can be variable but is clearly of critical importance. It is also unclear under which circumstances the expertise-based design should be the design of choice [3, 5–8].

The aim of this study, therefore, was to explore when the expertise-based approach would be considered an acceptable and feasible design. It examined the range of views that surgeons and methodologists have regarding the use of the expertise-based RCT design. Specific objectives were to explore surgeons’ and methodologists’ views on the acceptability and use of expertise-based trial designs; and to explore the circumstances in which such a design could or should be used. To our knowledge this is the first study to explore in-depth views on the use of an expertise-based design to compare surgical procedures.

## Methods

### Study design

The choice of research method was informed by the aim and objectives. As this study aimed to explore in-depth surgeons’ and methodologists’ perspectives regarding an expertise-based trial design, the use of semi-structured interviews was considered the most appropriate method for capturing subjective meanings and experiences [9–11]. Qualitative methods aim to provide an in-depth understanding by exploring and capturing phenomena from the perspective of those being studied, using methods that are sensitive to the study context. Qualitative methods are considered especially suitable for exploring new topics and for obtaining insightful data on complex issues and have been successfully used previously in the context of surgical trials [12]. The COnsolidated criteria for REporting Qualitative research (COREQ) Checklist has been used to ensure completeness of reporting items.

### Recruitment, sampling and consent

Purposive (with respect to professional background, surgical areas of familiarity and prior trial experience), and snowballing techniques [13, 14] were employed to derive the sample. Our final sample size was guided by the concept of data saturation. Initially, it was anticipated that between 10 and 15 participants would be sufficient to identify a range of experiences and views. It was not intended for this project to be exhaustive in terms of all views on the topic. At least five methodologists and five surgeons, respectively, were sought. Methodologists could come from any academic background but had to be involved in trial design in some way (e.g. statistician). No restriction was made on the surgical specialty. An initial list of potential participants was drawn up driven by knowledge of the surgical trial literature, personal involvement in surgical trials and related research projects and networks. Initial contact was either by email or face to face followed by an email with invitation letter. Recruitment was focused on UK-based participants to an extent driven by convenience and a preference for face-to-face interviewing and primary interest in the UK clinical setting. Participants were asked to suggest suitable names of further individuals who might be willing to participate. All participants gave written consent before participating in interviews. Some of the participants were personally known to the lead researcher prior to study conduct.

### Data collection

Interviews with surgeons and methodologists were conducted by one researcher (JAC) who had undergone formal training in qualitative methods (interviewing and analysis) and who had also received mentoring from experienced qualitative researchers. The interviewer has a background in trials methodology, was a senior trial statistician, and had been involved in designing and conducting surgical trials for 10 years post PhD. Interviews explored individual views about expertise-based designs and what role (if any) they saw for the design versus the standard within-surgeon approach. A topic guide was developed prior to the first interview and it was used to facilitate the interview conversations. It included specific questions tailored to methodologists, trial principal investigators and surgeons, respectively (Additional file 1). Opinions were sought on issues such as the ethics, practicality of the design, including whether they varied according to clinical area and research questions. Views regarding acceptability in the surgical community and influence of setting (e.g. NHS) were addressed. For surgeons, willingness to participate in a study with an expertise-based design was explored and correspondingly for methodologists, their willingness to use such a design.

### Data analysis

Interviews were audio-recorded and transcribed verbatim (transcripts were checked only by the interviewer). The analytical approach was both systematic and interpretive and the analysis was guided by a framework approach [15]. The framework approach is not aligned with a particular theoretical approach, but rather it is a flexible tool that can be adapted for use in qualitative studies that aim to generate themes inductively from data. A constant comparison approach was used to identify convergences and divergences across the data. Three authors (JAC, KG and ZS) familiarised themselves with the dataset and following initial familiarisation and group discussions, developed a coding framework based on a priori questions and emergent themes. Initial codes from this framework were systematically applied to the data. Data management and initial analytic coding was facilitated by the use of NVivo 10 software. The primary focus during the analysis was guided by the study aims set out in the protocol. During analysis, particular attention was paid to: the types of judgement and beliefs participants expressed in relation to expertise-based trial designs; personal willingness to use/participate in such a study, perceived advantages and disadvantages, practicalities and views of surgical community and patients. The analysis was finalised without any further input from the interviewees.

## Results

### Sample characteristics

Twelve interviews were conducted. No participants declined to participate and no participants dropped out without completing the interview. Interviewing was stopped after 12 interviews as there were no new themes emerging. The interviews took place between April and October 2013 and they lasted between 20 to 50 min. Eight were face to face (all in a research building setting) and four were by telephone with only the interviewer and the interviewee present. Of the 12 interviewees, eight were surgeons (S1–S8), four were methodologists (M1–3,5) (and one of the surgeons, S2, was also engaged in methodological research, indicated by M4). The surgeons were all (except for one registrar) consultants in the UK NHS; almost all of the surgeons worked at a teaching hospital. All 12 had experience of being involved in multicentre surgical trials and five had been the lead researcher (i.e. chief/principal investigator) of a randomised trial and were UK-based though some had worked in other countries. Three of the 12 participants were women, ages ranged from approximately 30 to 60 years old, and a number of participants were known by the interviewer prior to the interview through various professional connections. Most of the surgeons and methodologists had personally been involved in an expertise-based trial. Levels of clinical and research experience amongst the surgeons varied though most were of a senior professional level (consultant with substantial managerial responsibilities at clinical and/or academically and had been involved in multiple surgical RCTs) and reflected a range of surgical specialties. Methodologists were mostly very experienced in terms of professional track record (number of projects and publications).

### General views of an expertise-based trial design

Both the surgeons and the methodologists (with the exception of one surgeon who was noticeably more sceptical) expressed generally positive views regarding the potential of an expertise-based trial design. All the surgeons stated a willingness to be involved in principle with a trial with both expertise-based and standard designs. Methodologists were perhaps more agnostic in terms of its value but open to its use in particular situations:

‘You could imagine that there are different levels of expertise related to the two procedures, and what would provide a, you know, a fair comparison is I guess what we’re talking about.’ M1

Some methodologists expressed an explicit preference for running with a standard design if there was not a compelling argument to change:

‘I would go with the standard design because I think it – why make life more difficult than you have to?’ M3

‘But in some ways I still think you would start with (a standard design), could anybody be expert in both procedures at the same time, because it just takes out an extra potential factor that might bias your results, the surgeon being different.’ M1

While acknowledging potential advantages, the potential downsides to the use of an expertise-based design were also raised by both surgeons and methodologists: which included a view that the standard within-surgeon design should be the default; that the expertise-based approach has additional potential for biased findings; and that delivering only one procedure does not address all of the aspects of the trial that may be problematic for a surgeon. The potential advantages and disadvantages of using an expertise-based trial design over a standard trial design mentioned by participants are summarised in Table 1.

**Table 1:** Participants’ perceived potential advantages and disadvantages of an expertise-based versus a standard trial design

Expertise-based versus standard trial design	
*Advantages*	
Greater accommodation of surgeons’ treatment preferences	
Treatments performed in their ‘best light’	
More appealing to patients	
Better suited to some clinical settings	
*Disadvantages*	
Added complexity in terms of site set and administration, including greater co-ordination between surgeons required	
Design specific challenges which need to be addressed (e.g. defining an expert)	
Impact upon the patient-surgeon relationship	
Relation to clinical practice	
Perception of stakeholders	

### Potential advantages to an expertise-based design in practice

From the data it was apparent that interviewees identified four main reasons why expertise-based trial designs might be perceived as being advantageous over more standard approaches. These related to perceptions that (a) surgeons tend to hold particular preferences for (and/or have particular skills in) certain procedures; (b) treatments should be performed in their ‘best light’ within trials; (c) the design could be appealing to patients and (d) it could fit the clinical setting more appropriately. These are discussed in turn below.

- *Surgeons’ treatment preferences*

Firstly, several of those interviewed made the point that in principle expertise-based trial designs might prove more pragmatic and realistic compared with more standard approaches in the sense that surgeons very often have particular preferences for (and/or skills in) performing certain procedures – preferences and skills that expertise-based trials can usefully accommodate:

(There can be) ‘loads of different ways to do the same thing…we all have preferences and beliefs’. S6

‘Any RCT goes completely against the grain of being a surgeon. You’re a surgeon because you believe in something, you have an intervention to offer, you’re going to do your best to offer it, the best way you can.’ S8

‘I just don’t want to be doing that arm of it (the arm in which the surgeon has less experience). I think, you know, you can encompass a broader group of people into it (an expertise-based design) and that has some clear advantages. And … the primary drive for the surgeon is trying to do the best for the patient.’ S6

The expertise-based design could be more attractive to surgeons with a strong preference for a particular operation. It was stated that an expertise-based design could potentially help with ‘buy in’ (S7) from those with strong preferences, as long as the wider clinical team was willing to have ‘collective equipoise’– individually surgeons still get to do deliver what they wish to.

b) *Treatments would be performed in their ‘best light’*

Secondly, interviewees suggested that any treatments being investigated within a clinical trial setting should in theory be tested in their ‘best light’ (M2) in order to reflect best practice – something that again expertise-based trials are designed to accommodate:

‘… you get the best person doing the operation for the patient... So you’re not forcing a randomisation to where there’s expertise imbalance, and you’re also getting the surgeon who is really comfortable doing that particular intervention’ and, therefore, this design might be ‘more likely to show up a difference.’ M2

This idea was regarded by some as being particularly important for ensuring that crucial differences between procedures were not masked:

‘You probably are more likely to show differences between the two in that particular design (the expertise-based design), I suspect.’ M2

Surgeons who could only do one procedure could be involved in the study and, therefore, the results might also be more generalisable. This has a potential further advantage in terms of improving fidelity to allocation and avoiding ‘contamination’ (S5) between arms:

‘It is absolutely inevitable that the surgeons as they learn the new technique will change the way that they do their operations (specialising in one procedure).’ S5

c) *May be more appealing to patients*

It was also suggested that it was plausible that there could be a positive impact upon patient accrual as getting treated by someone who is considered an ‘expert’ could be appealing to some patients:

‘That’s an area that I’m not sure. Whether their perception …treated by an expert would increase accrual or not, (and) make it easier for the study from the patient perspective.’ M1

d) *May be better suited to the clinical setting*

In some settings doing only one operation might be practically and professionally more appealing as it can be scheduled on the same surgical list and operated on by the same surgeon.

‘I’m very happy doing X but very happy doing Y…. around the country in some units there are surgeons who don’t do Y, who just do X and they have colleagues in the same unit who do Y knees. And so to make it available to everyone, to buddy-up as a team is quite a good option.’ S6

### Disadvantages to an expertise-based design

Stated disadvantages of an expertise-based design were (a) the additional complexity in terms of set-up and recruitment; (b) the potential impact upon the patient-surgeon relationship; (c) how the model relates to clinical practice; and (d) potential negative perceptions of key external stakeholders.

- *Added complexity*

The complexity of the set up and administration beyond a standard design was seen as the main drawback by surgeons and methodologists:

‘I just think because you’ve added complexities in terms of the recruiting of the patients.’ (expertise-based more complicated over standard trial design) M5

With that in mind, the tide against it (setting up an expertise-based trial) is the complexities of setting it up and actually administering it, from a methodological point of view, and potentially getting people involved it sounds really good, but when you come to do it it’s not so straightforward.’ M2

This included reorganising the lists in some settings and also the impact of surgical waiting times:

‘….waiting lists for surgeons are often very different, and then their timing and if you’re getting randomised to you know, an X, but the X (operation) surgeon’s waiting list and slots are different to the Y or the Z (operations), then you could have you know, you’re in less control because the surgeons are independent in many ways working, and so there, methodologically there are issues there.’ M1

It was also noted that not having a team set up at sites could be problematic and also that certain settings, e.g. emergency procedure where there would only be one surgeon makes an expertise-based design more challenging:

‘I could certainly see in institutions where you don’t necessarily have (a team), it depends on the end relationship between colleagues and the communications and the politics of the environment as to whether or not this sort of thing (setting up an expertise-based trial) was straightforward.’ S4

In some settings, patients are not directed to a surgeon who could do the operation sooner even though that would be more efficient:

‘You will have a crazy situation where tomorrow the surgeon operating tomorrow hasn’t got cases, but the one who is not operating has got 20 patients waiting.’ S1

An expertise-based trial design was also noted to create another layer of complexity if the surgeon is involved in the recruitment and treatment pathway:

‘Whereas as soon as you start making it difficult and complicated for busy surgeons to take part in the trials, they just lose heart, and given the choice between doing a very well-paid commercial trial, or doing a more interesting but hard-work and financially unrewarding investigator-led academic trial, they’ll do the commercial activity.’ S7

While a surgeon might be more willing to take part if they only deliver one operation they still have to consider how they would recruit someone to a study where they might get the other:

‘There is then the question about how they can recruit into that study if they think one of the arms is ridiculous. So they have to work out where they sit on that ethically.’ S6

However, there was a perception that site set-up that is sensitive to the local situation, carried out with the expertise-based trial design in mind, and where there is a local willingness to modify processes for the trial can mitigate these added difficulties:

‘So when I go approach a unit I don’t approach an individual to come into a trial, I ask units to come into a trial.’ S5

‘How they (the complexities of expertise-based designs) are successfully addressed depends on the will of the individuals involved, so if you’ve got very informed surgeons who are keen on the trial and want to do it, it’s relatively easy.’ M2

b) *Impact upon the patient- surgeon relationship*

A key challenge was perceived by most but not all surgeons to be the relationship between the patient and the surgeon which for elective operations begins when the patient initially discusses having surgery. The perception of one surgeon was that maintaining this link was clinically preferably but also important to patients:

‘And all of that bit about the decision making as to whether they would have the operation – any operation. And if that patient then goes on to see another surgeon to have the operation is there some loss of confidence in the fact that they’ve now got another person to deal with. So it slightly challenges the gold standard clinical care pathway…’ S4

‘So if I were going to have a XXXX (operation), I would want to meet the surgeon beforehand. I’d want to have the operation, that guy helps in my shared decision. He does the operation, he follows me up and he looks after me if it goes wrong or if it does well.’ S4

Another interviewee noted that this had led to one of the surgeons dropping out of a previous expertise-based trial when they came to realise the implications regarding treating a patient assessed for surgery by another surgeon.

It was noted by one surgeon that some patients may not be familiar with the reality that surgeons specialise, and have expertise, in only one of the candidate operations and this would need to be raised if undertaking a trial with an expertise-based design:

‘Not all patients may understand that not all surgeons can do all of the operations. And that in itself is an interesting concept that you have to be able to deal with and how patients would react to that.’ S4

c) *Relation to clinical practice*

While acknowledging the potential advantages of adopting expertise-based trial designs in principle, several interviewees made the point that in clinical practice, surgeons might be regularly providing different procedures, some of which they will be better or worse at performing than others. Therefore, one could argue that to be really pragmatic these types of trial designs might not be the best approach and that more standard approaches are likely to better reflect normal practice at least in some settings:

‘All surgeons can do both and I’m perfectly happy with that because that follows clinical practice.’ S3

‘So I’m doing both operations and I’m fairly happy from all the discussions we’ve had when the trial was being initiated and started...’ S6

d) *Perception of stakeholders*

It was noted by one surgeon that methodologists and funding panels were a barrier to conducting expertise-based trials as they were perceived to prefer the standard design:

‘So, and persuading methodologists and panels that actually those biases they are worried about are trivial compared to the real life of biases in treatment and persuading the funding committees that they need to ignore such concerns is, I think is a challenge.’ S5

A more general concern raised by surgeons and methodologists was that they wanted to be involved in studies that would change practice and influence key bodies:

‘The question is, what is going to be the evidence that expertise-led trials are going to produce, which are going to allow NICE, for example, to make recommendations?’ S8

‘And the other issue about it is I would want a trial where the results can be used in the real world, and it worries me that if you have an expertise design that might limit its subsequent uptake of the interventions.’ S2/M4

### When to use an expertise-based trial design

In discussing when, and in what circumstances, they would consider expertise-based designs to be appropriate, interviewees stated that (1) study designs should be driven by the research question, (2) the extent to which the treatment comparators are perceived as being different and (3) the willingness/ability of surgeons to deliver both:

- *Study designs should be driven by the research question*

Several interviewees made the observation that study designs should be driven by the nature of the research questions and that if it is considered to be the best design from a methodological point of view then it should be used (regardless of whether it might not be as easy to implement as a standard RCT):

‘The main driver is that the question that was needing to be answered by the community was X (operation) versus Y (operation). But nobody, or almost no surgeon was prepared to do both procedures.’ M1

b) *The extent to which the treatment comparators are perceived as being different*

Interviewees also argued that the appropriateness or otherwise of expertise-based designs is likely dependent on the nature of the comparison interventions. For example, if it is a specialist/risky procedure then an expertise-based design might be more appropriate, whereas some comparisons might not be so suitable – as would be the case where there were only minor differences between procedures:

‘...(where the new procedure involves) little tweaks and way to do procedures, and in those cases I don’t think there is a role for an expertise-based trial in those settings.’ M1

c) *The willingness/ability of surgeons to deliver both*

As before, interviewees also made the point that expertise-based designs might prove particularly attractive to those surgeons who are not in equipoise and therefore not willing (or not able because of skill variation) to deliver both procedures:

‘ You know there are … surgeons who can do one procedure but they can’t do the other.’ S3

‘...(if you have a surgeon that says) “Well I can only do one of these. I’ve always done it. I believe in it.” Then that’s your perfect expertise candidate.’ S4

However, it was noted that if all surgeons were equally competent, then a standard trial design would be more appropriate**:**

‘So in the study they do both procedures, so they’re competent at both.’ S7

‘All surgeons can do both and I’m perfectly happy with that because that follows clinical practice.’ S3

### Methodological challenges to delivering expertise-based trials

A number of methodological issues were raised which would need to be addressed when a expertise-based trial design is used which were (a) the task of defining *expertise*; and (b) the possibility of bias resulting from different surgeons and related statistical clustering of outcome. These could also be viewed as disadvantages in that they are additional challenges over using  a standard design.

- *Defining who is an expert*

Exactly how to define expertise was raised as a challenging methodological issue by most interviewed and by both methodologists and surgeons. For example, should it be judged by the grade of a physician or by the numbers of procedures performed? Some argued that this could prove problematic particularly as it was suggested that some surgeons have a tendency to inflate numbers of procedures performed and/or deflate complications:

‘People inflate the numbers (of operations performed) and deflate their complications.’ S2/M4

The point was also made that regularly performing procedures does not necessarily equate with someone being more skilled:

‘And some people get very good after 10, and other people it takes 40 …’ S2/M4

Finally, the potential pejorative connotation of the implied term ‘expert’ implied by the name expertise-based trial was noted by two surgeons, i.e. being only in one arm they were not ‘expert’ in the other arm.

b) *Potential differences in surgical skill between surgeons in the randomised groups*

Also raised was the potential for bias in the differences between surgeons that might lead to systematic differences between the groups which are not purely due to the treatment:

‘But in some ways I still think you would start with, could anybody be expert in both procedures at the same time, because it just takes out an extra potential factor that might bias your results, the surgeon being different.’ M1

If the surgeons carrying out one arm were systematically different from the other arm this could lead to a difference being observed due to the surgeons and not the procedure.

An implication noted by multiple interviewees (surgeons and methodologists) delivering the treatments was the potential impact of statistical clustering of outcome around surgeon (this is that the outcome of operations conducted by the same surgeon are more similar than those conducted by different surgeons). This can occur in an expertise-based trial and is akin to a cluster randomised trial:

‘Okay, we need to allow for the inter cluster correlation of the operations within surgeon, that sort of thing, so there clearly has got to be some allowance for that in our calculations, from your trialists’ statistical point of view if you like. We had to address that particular aspect of it…’ M3

## Discussion

### Summary of work

To our knowledge, this is the first in-depth qualitative study of surgeons’ and methodologists’ attitudes to the use and conduct of expertise-based trials. It has helped to clarity the circumstances under which an expertise-based design could be identified as the design of choice and also revealed key insights into the perceived advantages and disadvantages of the design.

### When to use an expertise-based design

Our study suggested the surgical context to be particularly key to making a choice between alternative trial designs. The limited number of surveys of surgical opinion which have specifically addressed views on different design for a specific trial scenario also supports this [6, 7, 16]. There was general, though not exclusive, support for the standard design being the default option. The decision about when to use an expertise-based design was considered to vary between surgical contexts, there were certain key factors that would help inform that decision. These factors included the nature of the operations being compared (is it reasonable to expect one individual to do both operations – if not then an expertise-based trial might be preferable), and also who delivers each in practice (a team-based service delivery model lent itself more naturally to an expertise-based model). The participating surgeons reflected a broader range (and generally more positive) set of views on the desirability of using an expertise-based design than the methodologists. The latter appeared to be more concerned about the potential disadvantages of the design (e.g. differences in surgeons delivering the respective trial surgical procedures) which they felt required to be offset. Benefit for the surgeon or their colleagues (via greater continuity with their usual practice and preference) was perhaps more obvious to the former.

The expertise-based design was also seen as particularly useful in aiding situations where there might not be individual surgeon equipoise but rather where ‘collective’ or professional equipoise was present. The importance of physicians and surgeons being able to continue to take part in randomised trials when community rather than individual equipoise is dominant has been discussed widely in the literature [17] and the expertise-based design was viewed as particularly useful in this regard. This is consistent with the work of Devereux, Scholtes and colleagues [1, 18] who noted the particular strength of the expertise-based trial design in this regard. Such a benefit would not remove the generally challenging nature of recruiting to trials for clinicians with treatment preferences [19–21] as highlighted by a qualitative study which considered surgical trials with both expertise-based and standard designs [22].

### Advantages versus disadvantages of expertise-based versus other trial designs

A number of advantages were perceived with regards to the use of an expertise-based trial design. The potential attraction to surgeons of participating in a clinical trial while continuing to deliver surgery according to their preference was seen as particularly beneficial, and conducting the operation a surgeon was most comfortable with, experience in, and preferred was attractive. A number of these issues have been raised in previous opinion pieces [1, 18] and surveys of orthopaedic, urogynaecological and vascular surgeons [6, 7, 16] indicating that expertise can vary between operations and that in some settings an expertise-based trial design might be more appealing to, and better supported by, surgeons.

Our findings also suggested that the expertise-based design may also have other perceived benefits for patients. It was suggested that it could be attractive to patients enrolling within a trial that their surgeon would be an ‘expert’ in whatever procedure they were randomised to and would be delivering the procedure they were most comfortable with. However, others raised concerns about whether this design might result in a potential breach of the interpersonal relationship between recruiting surgeon and patient if, as a consequence of enrolling a patient in the trial, they would then hand over their care to a different surgeon. The importance of trust between the participant and health professional has high saliency in the literature around decisions to participate in trials [23–26] and it is, therefore, important that any impact of an expertise-based design on this domain is fully understood.

Further disadvantages noted by interview participants were the additional complexity of the site set-up requiring pairs or at least two sets, of surgeons to be available for the purposes of the trial. Previous expertise-based trials have been successfully conducted in a number of settings [3], though this has not always [27] been the case. However, it was suggested that is much easier in some settings or at least in these contexts this practical obstacle can be overcome [3]. The view that the likelihood of successful conduct is setting dependent was expressed by most of the interviewees in this study.

A number of methodological issues were raised including how to define *expertise*, the impact of the surgeon no longer being controlled for in the comparison, and the potential impact of clustering of outcome upon the required sample size. Administrative issues, such as the impact of the surgical waiting lists (that is substantial delays for receipt of surgery once its clinical need has been confirmed), or interacting with the health care system administration to schedule trial surgical procedures, was seen to be the most intractable problem where it was applicable, as it required multiple stakeholders to engage in a timely manner. Other issues (communication between surgeon’s delivering respective procedures) appeared to be thought of as solveable though potentially requiring more resources compared to an equivalent trial with a standard design. It is clear that these methodological issues require further investigation before the expertise-based design can be more widely adopted [28, 29]. More experience of conducting expertise-based trials in surgery is needed, along with in-depth evaluation of the impact upon set-up and delivery. Furthermore, critical evaluation of the practical implication of the different designs for addressing surgical procedure research questions across a range of surgical specialties is needed. Additionally, further work exploring the patient perspective on expertise-trial design would be beneficial.

### Strengths and limitations

To our knowledge this is the first in-depth qualitative study of the attitudes of surgeons and methodologists to the expertise-based trial design. While extensive work has been undertaken in other areas of trials methodology [12, 20, 22, 30] little depth work has been carried out to date regarding the potential use of some of the less common trial designs and particularly their use in the surgical context. This study also involved both surgeons and methodologists perspectives and included individuals from across a broad spectrum of practice which adds to the richness of the findings. The participants were also purposively chosen to have had experience of participating in, or leading, surgical trials. As such, our findings have been generated from an informed and experienced collective adding weight to the findings. This may, however, consequently suggest that the views may be less typical of the wider surgical community though possible more so of the research active sub-community.

A potential limitation of the study was that the participants were from the UK, which may limit the international insights generated by our findings (and may unduly reflect the practical constraints observed within the UK NHS particular within specific specialities). However, given that the primary focus of the study was on trial design features (rather than on direct implementation), many of the methodological issues raised will apply in all settings, e.g. the expertise-based design will need pairs, or two sets of surgeons to be available wherever that trial is conducted. A further limitation was the lead researcher’s relative lack of experience in using qualitative methods which may have limited the richness of the data collection. Furthermore, their understanding of both surgical trials and quantitative methodological concerns, while advantageous in terms of facilitating discussion of some more technical issues, could have inadvertently led to personal views being unconsciously transferred despite efforts taken to avoid this. The sample was also relatively small; however, as it is depth rather than breadth that is sought in qualitative studies, this sample size is not unusually so. Indeed, in terms of data saturation, we were satisfied that our sample size was appropriate and adequate for enabling us to sufficiently answer our research aims [13]. However, the study did not address patients’ perspectives which is a shortcoming, further studies to address this would be valuable.

## Conclusion

This study has shown that the expertise-based trial design is viewed as having significant potential to address known challenges with the design and conduct of surgical trials and has the potential to increased surgeon participation in trials under certain circumstances. In other settings the standard design was generally seen as the preferable design. Particularly suitable conditions for an expertise-based design include those where the surgical procedures under evaluation are substantially different, where they are routinely delivered by different health professional/surgeons with clear proficiencies in each; and contexts in which a multiple-surgeon model is operating and the trust between the patient and surgeons can be suitably protected. The standard trial design was, however, seen by most participants as the default design. Several logistical and methodological concerns remain to be addressed before the expertise-based design is likely to be more widely adopted.

## Additional file

Additional file 1: Topic guide – methodologists and surgeons. (DOCX 12 kb)

## Abbreviations

COREQ: COnsolidated criteria for REporting Qualitative research
NHS: National Health Service
RCT: Randomised Controlled Trial
