# Supplementary material for: Surgeons’ and methodologists’ perceptions of utilising an expertise-based randomised controlled trial design: a qualitative study
Source: Trials. 2018 Sep 6;19:478. doi: 10.1186/s13063-018-2832-z (PMC6127897; doi:10.1186/s13063-018-2832-z)
Supplement: Supplementary file 1 — Topic guide – methodologists and surgeons. (DOCX 12 kb) [file 13063_2018_2832_MOESM1_ESM.docx]

**Additional file 1**

**Topic guide – methodologists & surgeons**

- Personal involvement in surgical trials including whether the interviewee has been involved in a study which has used an expertise-based trial design
- Perceived benefits/flaws of an expertise-based trial design
- Views on the practicalities of an expertise-based trial design in their practice, centre and amongst their community
- Degree of willingness to personally take part in a study using an expertise-based trial design in the future and any preference regarding study design
- Degree of willingness to take use an expertise-based trial design to answer a research question addressing a surgical intervention [Methodologist/Surgeon chief investigators only]
- Views regarding acceptability to patients [Surgeons only]
- Views regarding feasibility of timing of consent and randomisation
- Views regarding the feasibility of an expertise-based trial design amongst the relevant surgical community, and whether it varies according to the clinical area and research question [Surgeons only]
